# SESOTHO trial (“Switch Either near Suppression Or THOusand”) – switch to second-line versus WHO-guided standard of care for unsuppressed patients on first-line ART with viremia below 1000 copies/mL: protocol of a multicenter, parallel-group, open-label, randomized clinical trial in Lesotho, Southern Africa

**DOI:** 10.1186/s12879-018-2979-y

**Published:** 2018-02-12

**Authors:** Alain Amstutz, Bienvenu Lengo Nsakala, Fiona Vanobberghen, Josephine Muhairwe, Tracy Renée Glass, Beatrice Achieng, Mamorena Sepeka, Katleho Tlali, Lebohang Sao, Kyaw Thin, Thomas Klimkait, Manuel Battegay, Niklaus Daniel Labhardt

**Affiliations:** 1 Clinical Research Unit, Department of Medicine, Swiss Tropical and Public Health Institute, Socinstrasse 57, 4051 Basel, Switzerland; 2 University of Basel, 4051 Basel, Switzerland; 3 Division of Infectious Diseases and Hospital Epidemiology, University Hospital Basel, 4051 Basel, Switzerland; 4 SolidarMed, Swiss Organization for Health in Africa, Maseru/Butha-Buthe, Lesotho; 5 Butha-Buthe Government Hospital, Butha-Buthe, Lesotho; 6 District Health Management Team Butha-Buthe, Butha-Buthe, Lesotho; 7 Research Coordination Unit, Ministry of Health of Lesotho, Maseru, Lesotho; 8 Molecular Virology, Department of Biomedicine, University of Basel, 4051 Basel, Switzerland

**Keywords:** HIV, Viral suppression, Treatment failure, First-line antiretroviral therapy failure, Switch to second-line antiretroviral therapy, Lesotho, Southern Africa, Randomized controlled trial, Low-level viremia

## Abstract

**Background:**

The World Health Organization (WHO) recommends viral load (VL) measurement as the preferred monitoring strategy for HIV-infected individuals on antiretroviral therapy (ART) in resource-limited settings. The new WHO guidelines 2016 continue to define virologic failure as two consecutive VL ≥1000 copies/mL (at least 3 months apart) despite good adherence, triggering switch to second-line therapy. However, the threshold of 1000 copies/mL for defining virologic failure is based on low-quality evidence. Observational studies have shown that individuals with low-level viremia (measurable but below 1000 copies/mL) are at increased risk for accumulation of resistance mutations and subsequent virologic failure. The SESOTHO trial assesses a lower threshold for switch to second-line ART in patients with sustained unsuppressed VL.

**Methods:**

In this multicenter, parallel-group, open-label, randomized controlled trial conducted in Lesotho, patients on first-line ART with two consecutive unsuppressed VL measurements ≥100 copies/mL, where the second VL is between 100 and 999 copies/mL, will either be switched to second-line ART immediately (intervention group) or not be switched (standard of care, according to WHO guidelines). The primary endpoint is viral resuppression (VL < 50 copies/mL) 9 months after randomization. We will enrol 80 patients, giving us 90% power to detect a difference of 35% in viral resuppression between the groups (assuming two-sided 5% alpha error). For our primary analysis, we will use a modified intention-to-treat set, with those lost to care, death, or crossed over considered failure to resuppress, and using logistic regression models adjusted for the prespecified stratification variables.

**Discussion:**

The SESOTHO trial challenges the current WHO guidelines, assessing an alternative, lower VL threshold for patients with unsuppressed VL on first-line ART. This trial will provide data to inform future WHO guidelines on VL thresholds to recommend switch to second-line ART.

**Trial registration:**

ClinicalTrials.gov (NCT03088241), registered May 05, 2017

## Background

The management of first-line antiretroviral therapy (ART) failure is a crucial step to achieve the third pillar of the UNAIDS 90–90-90 targets, namely viral suppression in 90% of all HIV-positive individuals on treatment, and ensure successful treatment outcomes [1]. The World Health Organization (WHO) recommends viral load (VL) measurement as the preferred monitoring strategy for HIV-infected individuals on ART in resource-limited settings [2, 3]. The current WHO and Lesotho guidelines define virologic failure as two subsequent VL ≥1000 copies/mL despite good adherence [2–4]. Specifically, the guidelines recommend that in case of a VL ≥1000 copies/mL, the patient should undergo enhanced adherence counseling, and a second VL test should be performed 8–12 weeks later. A second VL ≥1000 copies/mL despite good adherence triggers switch to an appropriate second-line regimen, whereas if VL returns to < 1000 copies/mL, first-line ART is continued unaltered. Thus, the WHO guidelines define patients with a VL below 1000 copies/mL as “non-failures”. However, the guidelines acknowledge that the threshold of 1000 copies/mL is based on low-quality evidence and, in fact, the optimal threshold for defining virologic failure and for switching ART regimens has not been established. In contrast, European [5] and North American [6] guidelines suggest to switch at much lower thresholds, namely above 50 and 200 copies/mL, respectively.

Several observational cohort studies and systematic reviews from resource-rich and resource-limited settings have demonstrated the emergence of major drug resistant mutations (DRMs) and a high risk of virologic failure among patients with sustained low-level viremia [7–16]. A recently published study from our research consortium in Lesotho supports these findings, showing that 94% (17/18) of patients with VL between 80 and 999 copies/mL harbored critical DRM against at least two drugs of the current first-line regimen. In this small patient subgroup with VL between 80 and 999 copies/mL, who were continued on first-line ART as per national guidelines, none achieved viral resuppression one year later [17, 18].

In high-income settings with easy access to VL monitoring, individualized patient management and a “watch-and-wait” strategy with monthly monitoring for patients with low-level viremia may be appropriate [5, 6]. However, if patients in resource-limited settings with a VL below 1000 copies/mL are defined as “non-failures”, they will not receive any follow-up VL for at least 6 to 12 months – depending on the frequency of VL monitoring recommended in national guidelines and on the availability of VL in a given setting – and may thus continue a failing regimen for considerable time. While the risk of onward HIV transmission and disease progression seems to be low in patients with low-level viremia [19–21], defining patients with VLs below 1000 copies/mL as “non-failures” in resource-limited settings, such as Lesotho, may bear a considerable risk from an individual as well as public health perspective as progression to full failure with high viremia will remain undetected for months.

To our knowledge there are no published or ongoing randomized controlled trials (RCTs) from resource-limited settings assessing VL thresholds for switch to second-line ART, as summarized in the recent viewpoint of Ellman et al. in the Journal of the International AIDS Society [22]. This manuscript is the study protocol of the SESOTHO (“Switch Either near Suppression Or THOusand”) trial. The SESOTHO trial challenges the current WHO guidelines, assessing whether a threshold of 100 copies/mL compared to the WHO-defined threshold of 1000 copies/mL for switching to second-line ART among HIV-positive patients on first-line ART will lead to higher viral resuppression rates.

## Methods

### Setting and design

The SESOTHO trial will be conducted in the district of Butha-Buthe, in northern Lesotho, Southern Africa. Lesotho has an estimated adult HIV prevalence of 25% [23]. Little is currently known regarding the third UNAIDS target (viral suppression in 90% of people on ART) in Lesotho as routine VL monitoring has been introduced only recently. Butha-Buthe is a rural district with an estimated 110,000 habitants, who are mostly subsistence farmers or mine workers, or construction or domestic workers in neighboring South Africa. According to the Demographic Health Survey 2014, the adult HIV prevalence in the district is 21.2% [23].

Since the beginning of 2016 the district hospital of Butha-Buthe offers VL testing, thanks to a close collaboration between the Ministry of Health of Lesotho, the Swiss Tropical and Public Health Institute (Swiss TPH), Department of Biomedicine University of Basel and SolidarMed, Swiss Organization for Health in Africa. All patients on ART in Butha-Buthe district have access to routine VL monitoring and VL results are stored in an encrypted and password-protected online database (https://visibleimpact.org/projects/1261-molecular-hiv-monitoring-in-lesotho).

This is a multicenter (2 hospitals [Butha-Buthe Government Hospital and Seboche St Charles Mission Hospital] and 10 rural health centers), parallel-group (1:1 allocation), open-label, superiority, prospective RCT in a resource-limited setting.

### Screening, randomization and interventions

**Fig. 1:**
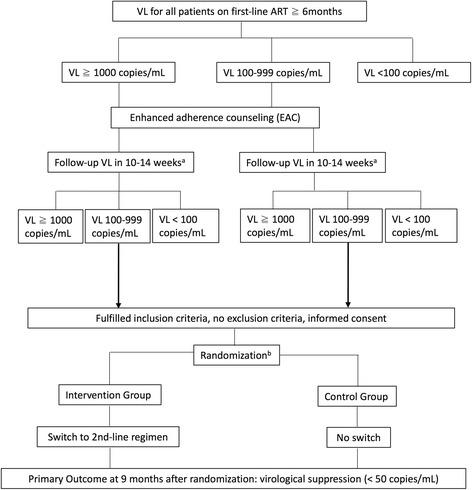
Flow Chart of the SESOTHO trial: screening, randomization and primary endpoint. The first part of the flow chart (incl. EAC and follow-up VL) represents routine ART monitoring in the study district. ^a^ 10–14 weeks after the time point the patient has been informed. ^b^ stratified by centres, demographic groups and VL level at enrolment

Patients will be screened for eligibility according to the criteria in Table 1 by the study physician using the above mentioned online database with all VL results from the participating health facilities in Butha-Buthe district. Figure 1 displays the flow chart with the screening and randomization process.

**Table 1:** Eligibility criteria for the SESOTHO trial

Inclusion Criteria	Exclusion Criteria
a) On non-nucleoside reverse transcriptase inhibitor (NNRTI) based ART (standard first-line regimen in Lesotho) for at least 6 months b) Two consecutive VL ≥100 copies/mL, with the latest VL between 100 and 999 copies/mL c) Participant lives and/or works in the district of Butha-Buthe and declares to seek follow-up at one of the study-facilities d) Signed written informed consent. For children aged < 18 years and illiterate patients, a literate caregiver or witness, respectively, must provide written informed consent (see Ethical considerations).	a) On ART less than 6 months b) On protease inhibitor (PI) or integrase inhibitor containing ART regimen c) Poor adherence (self-reported at least 1 dose of a once-daily regimen missed in the last 4 weeks, respectively two doses of a twice-daily-regimen) d) Clinical WHO stage 3 or 4 at enrolment

Eligible patients will be invited to join the study, and informed consent will be sought from the patient by the study physician and a study nurse who speaks the local language. Eligible and consenting patients will be randomized by the study physician in a 1:1 allocation, using sealed, opaque, and sequentially-numbered envelopes, stratified by centres (hospitals; health centres), demographic group (adults; children <16y; pregnant women) and level of VL at enrolment (100–599; 600–999 copies/mL) with randomly-varying block sizes. The randomization list was generated by the trial statistician and the envelopes were prepared by persons independent from the trial. According to their randomized allocation, participants will either be immediately switched to a second-line ART regimen (intervention group) or remain on first-line ART (control group, standard of care). Choice of second-line ART is based on the national ART guidelines [4].

### Primary endpoint

The primary endpoint is viral resuppression (VL < 50 copies/mL) 9 months after randomization. The rationale for this time-point is that, according to national guidelines, VL should be measured 6 months after switch to second-line ART. If this VL is above 1000 copies/mL, enhanced adherence counseling is introduced and a follow-up VL is performed 3 months later [4]. Hence, 9 months is the earliest possible time point for virologic failure (versus viral resuppression) after switch to second-line ART. Moreover, using 9 months instead of 6 months takes into account that PI-based regimens might need more time to achieve viral resuppression.

### Secondary, exploratory, and subgroup endpoints

These endpoints are outlined in Table 2. We defined one pre-specified subgroup: Individuals showing a > 0.5 drop in log_10_ VL from the first screening VL measurement (before enhanced adherence counseling) to the second screening VL measurement (i.e. VL at enrolment). According to WHO guidelines [2], these individuals would not be switched to second-line ART regardless of their absolute VL level. However, such patients are considered eligible for inclusion in this trial, and hence they could be randomized to either treatment arm. The rational for this decision is based on results of our previous study [17]. In that study, all patients with a > 0.5 drop in log_10_ VL and a second VL between 100 and 999 copies/mL harboured HIV with relevant drug resistance mutations, indicating a need for switch to second-line ART despite the large drop in VL after enhanced adherence counseling. We assume that individuals showing a > 0.5 drop in log_10_ VL but still with VL ≥100 copies/mL will benefit from switching to second-line ART. Therefore, we hypothesize the same outcome (significant difference in viral resuppression between the two treatment arms) in this subgroup.

**Table 2:** Secondary, exploratory and subgroup endpoints of the SESOTHO trial

	Definition	Time point following randomization	Remarks
Secondary endpoints			
VL level	Proportion of participants with different levels (VL < 100, < 200, < 400, < 1000 copies/mL)	9 months	
VL at 6 months	Proportion of participants with viral resuppression (< 50 copies/mL)	6 months	
Sustained virologic failure	Proportion of participants with unsuppressed VL > 50 copies/mL at 6 and 9 months	6 and 9 months	
Adherence	Proportion of participants with good adherence	3, 6, and 9 months	Definition of “good adherence”: self-reported no dose missed of a once-daily-regimen, respectively less than two doses of a twice-daily-regimen, during the last month
Clinical outcomes	Change in values (versus values at baseline) of body-weight (kg), CD4-cell count (cells/μL), haemoglobin (g/dL), lipids (total cholesterol, LDL, HDL, triglycerides; mmol/L); proportion of patients with newly-recorded clinical WHO-stage 3 or 4 events; proportion of patients died (all-causes)	9 months	
(Serious) Adverse Events	Proportion of patients with Adverse Events (AE) or Serious Adverse Events (SAE)	9 months	AE and SAE are graded according to Common Terminology Criteria for Adverse Events (v4.0, published May 28, 2009)
Long-term follow-up	Proportion of patients that are alive, retained in care and virologically suppressed	24 months	Definition of “virologically suppressed”: < 50 copies/mL
Potential effect modifiers			
Primary endpoint by demographic groups	Viral resuppression by adults vs children vs pregnant women	9 months	Definition of “viral resuppression”: < 50 copies/mL
Primary endpoint by baseline VL groups	Viral resuppression by baseline VL 100–599 vs 600–999 copies/mL	9 months	Definition of “viral resuppression”: < 50 copies/mL
Exploratory endpoints			
Cost-effectiveness	Staff costs (clinical and laboratory); costs of ARVs; costs of drugs for prevention of opportunistic infections and other concomitant medications; laboratory costs (CD4 cell count, VL, blood chemistry, blood count and other diagnostic procedures); estimation of health impact/benefits outcomes (e.g. DALY)	Between baseline and 9 months and 24 months (long-term follow-up)	
Drug resistance mutations	Prevalence of major drug resistance mutations a) on all baseline VLs and b) on all VLs that remain unsuppressed (> 50 copies/mL) at 9 months for all samples for which an RT-PCR amplification is successful	At baseline and at 9 months	Definition of “major drug resistance mutations”: Stanford University HIV Resistance Database (http://hivdb.stanford.edu)
Subgroup endpoint			
Log-drop	Viral resuppression among individuals with a > 0.5 drop in log10 VL between the first screening VL and the second screening VL (i.e. VL at enrolment)	9 months	Definition of “viral resuppression”: < 50 copies/mL

### Data collection and management, biologic material, and follow-up

All data collected during the scheduled visits will be recorded on paper case report forms and subsequently entered into a password-protected database using EpiData (v4.0.2.101). Access and type of activity for an individual is regulated by privileges assigned to his/her user identification code and password. Data will be double-entered, and modifications are retrievable for reviewing. Apart from the informed consent form, all study documents will not contain any names but solely the study-ID. There is one master list with the subject identification code, which will be stored in a password-protected, encrypted online cloud and only accessible for pre-defined study personnel. Participant files will be maintained in storage for a period of at least 10 years after completion of the study.

**Fig. 2:**
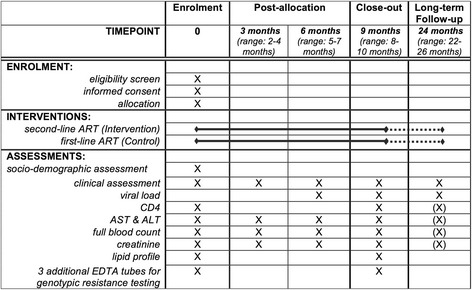
SPIRIT Flow Diagram of the SESOTHO trial

Participants will undergo phlebotomy at recruitment, 3, 6, 9, and 24 months after randomization. For each participant, study-ID-coded blood samples will be stored at − 80 °C at the laboratory of Butha-Buthe hospital. All samples collected fall under the biobank agreement, accepted by the EKNZ (EKNZ BASEC UBE-Req. 2016–00708) and the ethics committee in Lesotho (ID134–2016). VL determination will be done on COBAS TaqMan® HIV-1 Test, v2.0 (Roche Diagnostics) with reliable lower limit of detection of 20 copies/mL following the manufacturer’s protocol for VL analysis. Figure 2 displays the SPIRIT flow diagram with the overview of data collection, laboratory assessments and follow-up visits. In both groups, follow-up is in-line with Lesotho guidelines [4] with additional laboratory testing at 3 and 9 months.

### Sample-size

Studies assessing virologic outcomes in patients with a VL < 1000 copies/mL in resource-limited settings are scarce. Most studies found viral resuppression rates after switch to second-line of 60–80% (with various definitions of viral resuppression levels and time points) [24–28]. Due to the limited published data, our sample size assumptions were primarily based on our own data from a previous cohort study in Lesotho, although the sample size of that study was small and therefore the confidence intervals of our estimates were wide [17]. Based on those data, we expect 9-month viral resuppression rates of 25% in the control arm and 60% in the intervention arm, assuming a lost-to-follow-up (LTFU) rate of 10% in each arm, and counting participants who are LTFU as failures, i.e. not virologically resuppressed. Assuming a two-sided type 1 error of 5%, 80 individuals (40 per group) are needed to detect a 35% difference with 90% power. This sample size provides 78% power to detect a 30% difference. With an estimated 10% trial participation refusal rate, we need to screen approximately 90 participants.

### Analyses

Analyses will be performed following CONSORT guidelines [29] and a modified intention-to-treat principle including all participants as randomized, but LTFU, death (all-causes), and change in regimen line prior to determination of the primary endpoint considered as failures. Change in regimen line is defined as a change to a new drug class (i.e. from a NNRTI to a PI). Substitutions within same drug class (i.e. changing from tenofovir to zidovudine because of renal impairment) will not be considered as a “change in regimen line”. We will perform a sensitivity analysis (per protocol set) including only participants who finished the 9 months according to the protocol (that is, alive, retained in care, no change in regimen line other than that indicated by the randomization, and VL measurement available at 9 months). We will present a CONSORT flow chart of the participants, including screening, enrollment and follow-up. Baseline characteristics will be presented by randomized groups using appropriate summary statistics; no formal testing will be performed [30]. Outcomes will be summarised at all follow-up visits by randomized groups using appropriate summary statistics. Analysis of the primary endpoint will use a logistic regression model adjusted for the randomisation stratification factors (centers, demographic groups, and VL at enrolment, as detailed above). For analyses of other binary outcomes and continuous outcomes we will use similar logistic and linear regression models, respectively. Potential effect modifiers (as shown in Table 2) will be assessed by incorporating the appropriate interections into the regression models.

All analyses will be performed using R (the R Foundation for Statistical Computing) or Stata (version 14, Stata Corporation, Austin/Texas, USA). For all tests, we will use two-sided *p*-values with alpha 0.05 level of significance. A separate detailed data analysis plan will be developed, including for the cost-effectiveness analysis.

### Monitoring, auditing, and data safety and monitoring board (DSMB)

A minimum of two external monitoring visits will assess adherence to the trial protocol, accuracy of completed case report forms, and the electronic dataset. The Pharmaceutical Medicine Unit of the Division Medicines Research (MedRes) at the Swiss Tropical and Public Health Institute, which acts as an independent academic contract research organization, will perform the monitoring. The Principal Investigator agrees to allow inspectors from regulatory agencies to review records and will assist the inspectors in their duties, if requested.

Given that the recruitment is projected to happen fairly quickly, determination of the primary endpoint is after 9 months, safety profiles of all drugs are well-known, and the intervention does not include any new drugs, a DSMB will not be installed.

### Ethical considerations

This trial has been approved by the National Health Research and Ethics Committee of the Ministry of Health of Lesotho (ID48–2017, 05.07.2017) and the Ethics committee in Switzerland (Ethikkomission Nordwest- und Zentralschweiz; EKNZ BASEC UBE 2017–00201, 10.04.2017).

Prior to randomization, all participants will provide oral and written informed consent: The study nurse/physician will explain the information sheet to participants. Once all open questions have been clarified and upon agreement to participate, the participant will sign the informed consent form. Iliterate participants will provide a thumb-print and a witness (independent to the trial and > 21 years old), chosen by the participant, will co-sign the form. For children and young adults aged < 18 years, a literate caregiver > 21 years old (person that takes care of the child/young adult) will provide oral and written informed consent. The informed consent is provided in English and the local language, Sesotho, and the participant will receive a copy of the consent form in written format to take away. The participant has the right to withdraw at any time without giving reasons. In case of withdrawal, only data collected until the time of withdrawal will be used for research purposes (fully anonymized, identifyer removed). A separate, detailed safety monitoring plan has been developed to handle (S)Aes, in-line with swiss and lesotho ethics regulations. The study physician is responsible for all safety procedures. If a participant develops an adverse event of Grade 2 or higher at last study visit, he/she will remain under observation by the study physician even after study termination, until the adverse event is resolved or stabilized.

Participation in this study is not anticipated to cause any substantial additional risk or cost to the participant. Therefore we will not pay compensation to the participants. Free AirTime (local prepaid money for cellphone usage) will be provided to the study nurses/physicians for the duties of the study. We will consider a step-wise remuneration for study nurses/physicians per enrolled study participant.

### Trial status and recruitment

The trial has been launched at the two study sites with the highest patient load (Butha-Buthe Hospital and Seboche St Charles Hospital) and the first patient was recruited at Butha-Buthe Hospital on August 01, 2017. If necessary, recruitment will subsequently be rolled out to rural health centres. We anticipate identifying approximately 14 eligible participants/month and a 10% refusal rate, therefore we project a total recruitment period of 7 months to achive our target sample size of 80 patients.

## Discussion

By the end of 2016, an estimated 11.7 million individuals, representing 60% of all people living with HIV in Eastern and Southern Africa, were receiving ART [31]. In resource-limited settings, scaling up the number of people on ART relied strongly on the WHO public-health approach [32]. Standardized, clear and pragmatic guidelines allowed decentralization of care to less specialized health care facilities, such as nurse-led health centers [33]. This means that HIV care in such settings is often provided by less-specialized health personnel and so management usually strictly follows national ART guidelines, which in turn follow WHO guidelines. The 2013 WHO guidelines introduced VL monitoring as the preferred monitoring strategy for individuals on ART, even in resource-limited settings. Although implementation of VL monitoring is challenging, i.e. in laboratory infrastructure, technical capacities and affordability, data from 12 countries in Eastern and Southern Africa showed that about 44% of people on ART had access to VL monitoring and all countries but one in the region have established a national policy on routine VL monitoring [31]. With VL monitoring becoming available for millions of patients on ART in this region, it is important to reassess the WHO recommendations on virologic failure. Initially, WHO guidelines defined virologic failure with a threshold of 5000 copies/mL [34]. This threshold was lowered to 1000 copies/mL in the 2013 revised WHO guidelines and was maintained in recent consolidations in 2016 and 2017 [35]. However, the WHO guidelines and the Lesotho guidelines acknowledge that there is no clear evidence for supporting a threshold of 1000 copies/mL [2, 4]. In resource-limited settings, where care strictly follows national and WHO guidelines, the decision to define patients with sustained unsuppressed VL below 1000 copies as “non-failures”, and therefore continue with unaltered drug regimen, bears the danger that emerging virologic failures will remain unrecognized over unnecessarily long periods despite the availability of VL monitoring.

This trial has several limitations: The proposed switch to second-line at a lower threshold will lead to a higher number of people receiving second-line ART, which remains 2–3 times more expensive than first-line ART [36]. Moreover, current first-line therapy in Southern Africa usually consists of tenofovir disoproxil fumarate, lamivudine, and efavirenz, available as one pill once daily [37]. In contrast, second-line ART typically consists of ritonavir-boosted lopinavir, lamivudine, and zidovudine, a regimen consisting of two to three pills twice daily. A higher pill burden has been shown to reduce adherence [38]. While the current WHO threshold may be associated with switching too late, the threshold in the intervention group of the SESOTHO trial may, for some individuals, lead to unnecessary (or premature) switches, with higher cost to the health system and higher pill burden to the patient. To address these limitations we plan a) a cost-effectiveness analysis, and b) genotypic resistance testing on baseline samples, in order to determine how many patients were switched without detectable drug resistance mutations in both groups.

In summary, the SESOTHO trial aims to generate evidence to inform future WHO guidelines, regarding whether a lower VL threshold (e.g. at 100 copies/mL) for switching to second-line ART in resource-limited settings will result in higher rates of viral resuppression. To our knowledge this is the first and only ongoing RCT addressing the question of VL threshold for switch to second-line ART in Southern Africa.

## Abbreviations

(S)AE: (Serious) Adverse Events
ALT: Alanine Transaminase
ART: Antiretroviral Therapy
AST: Aspartate Transaminase
DRM: Drug Resistance Mutations
HDL: High Density Lipoprotein
HIV: Human Immunodeficiency Virus
LDL: Low Density Lipoprotein
LTFU: Loss-To-Follow-Up
MedRes: Medicines Research Division, Swiss TPH
NNRTI: Non-Nucleoside Reverse Transcriptase Inhibitor
PI: Protease Inhibitor
Swiss TPH: Swiss Tropical and Public Health Institute
UNAIDS: United Nations Programme on HIV/AIDS
VL: Viral Load
WHO: World Health Organization
